# Impact of Circadian Desynchrony on Spermatogenesis: A Mini Review

**DOI:** 10.3389/fendo.2021.800693

**Published:** 2021-12-16

**Authors:** Ferdinando Fusco, Nicola Longo, Marco De Sio, Davide Arcaniolo, Giuseppe Celentano, Marco Capece, Roberto La Rocca, Francesco Mangiapia, Gianluigi Califano, Simone Morra, Carmine Turco, Gianluca Spena, Lorenzo Spirito, Giovanni Maria Fusco, Luigi Cirillo, Luigi De Luca, Luigi Napolitano, Vincenzo Mirone, Massimiliano Creta

**Affiliations:** ^1^ Urology Unit, Department of Woman, Child and General and Specialized Surgery, University of Campania “Luigi Vanvitelli”, Caserta, Italy; ^2^ Department of Neurosciences, Reproductive Sciences and Odontostomatology, Urology Unit, University of Naples “Federico II”, Naples, Italy; ^3^ Department of Woman, Child and General and Specialized Surgery, Urology Unit, University of Campania Luigi Vanvitelli, Naples, Italy

**Keywords:** circadian desynchrony, circadian rhythms, clock genes, spermatogenesis, fertility

## Abstract

The purpose of this mini review is to provide data about pre-clinical and clinical evidence exploring the impact of circadian desynchrony on spermatogenesis. Several lines of evidence exist demonstrating that disruption of circadian rhythms may interfere with male fertility. Experimental knock-out or knock-down of clock genes, physiologically involved in the regulation of circadian rhythms, are associated with impairments of fertility pathways in both animal and human models. Moreover, disruption of circadian rhythms, due to reduction of sleep duration and/or alteration of its architecture can negatively interfere in humans with circulating levels of male sexual hormones as well as with semen parameters. Unfortunately, current evidence remains low due to study heterogeneity.

## Introduction

Globally, it has been estimated that infertility affects approximately 8-12% of couples, with a male factor being a primary or contributing cause in about 50% of couples (1, 2). Unfortunately, the cause of male infertility is unknown in about 30% of these cases (3, 4). Environmental endocrine disruptors, often consequences of human activity, have been widely investigated as agents potentially involved into the pathogenesis of infertility in animals and humans (5, 6). Over the past 30 years primary focus has been directed to the effects of chemicals environmental endocrine disruptors found in plasticizers, pharmaceuticals, and pesticides such as bisphenol A or glyphosate (7, 8). In recent years, however, the effects of non-chemical environmental endocrine disruptors such as that interfering with circadian rhythms (CR) leading to circadian desynchrony (CD) also gained growing interest in the pathophysiology of male infertility (5). The aim of this mini review is to provide the latest information on pre-clinical and clinical evidence about the relationship between CD and spermatogenesis.

## Methods

The authors conducted a literature search of available sources evaluating the pathophysiology and clinical evidence about the relationship between CD and impaired male reproduction, with a special focus on spermatogenesis. Web of Science, PubMed, and Scopus databases were searched to find relevant articles. The information found in the selected studies was carefully evaluated and it is described and discussed in the following sections.

### CR and CD: Definition and Pathophysiology

CR consist in daily oscillations in physiology processes (gene expression, metabolism, activity patterns and serum hormone levels) and behavior recurring with a 24h period. CR represent an ubiquitous feature in living organisms: they modulate function since unicellular life and gained an higher complexity multicellular organisms (9, 10).

In vertebrates, CR are hierarchically organized. They are both autonomous, based on cellular cycle which builds rhythmic activity of tissues, and controlled by synchronization through environmental signals (11). The main regulator of these rhythms is represented by the hypothalamic suprachiasmatic nucleus (SCN) (12). The cells in the SCN orchestrate rhythms in endocrine, physiological, and behavioral parameters through the activation of other central circadian oscillators (e.g., the hypothalamus and pituitary gland), with a period close to, but not exactly 24 hours (13, 14).

CR are strongly entrained by the daily photoperiod as they are influenced by the environmental light-dark cycle which has been reported to modulate the expression of several genes (15). Therefore, light is the most effective environmental synchronizing agent for the clock of human beings: it represents a direct drive to the nervous system through activation of intrinsic photosensitive retinal ganglion cells and transduced directly to the SCN through the retinohypothalamic tract (16), and an indirectly through the intergeniculate leaflet (17, 18).

Clock genes play a central role in orchestrating CR. In mammals, the main clock genes include: *Period* genes (*Per 1/2/3*, Period Circadian Regulator1/2/3), Circadian Locomotor Output Cycles Kaput (*Clock*) gene, *Cryptochrome* genes (*Cry 1/2*, Cryptochrome Circadian Regulator 1/2) *and* Aryl hydrocarbon receptor nuclear translocator-like protein 1 (ARNTL, also known as MOP3 or Bmal1) (19). Clock genes are involved in several physiological processes and diseases including ageing, metabolism, fertility, cardiovascular health, and cell proliferation (20, 21).

Throughout the last two centuries, modern lifestyles increasingly deprive us of natural zeitgebers (dt. time-givers) and technological advances have dramatically changed individual work and rest patterns (22–24). Electricity and constant accessibility to light, energy, food, the possibility to travel across time zones created an environment that is different from the one of the last two centuries. These environmental perturbances strongly contribute to the pathophysiology of CD, a condition defined for the first time by Sack & Lewy in 1997 as a specific type of circadian disruption occurring when endogenous rhythms become misaligned with daily photoperiodic cycles (25–27).

Growing evidence support the hypothesis that disruption of CR is involved in the pathophysiology of several diseases such as metabolic impairment, cardiovascular and sleep disorders, psychiatric illness, cancers such as urothelial carcinoma, and infertility (28–32).

### CD and Male Infertility

Physiological processes regulating fertility need to be appropriately synchronized with the external environment to guarantee reproductive success. In the testis several models of temporal organization had been found. The complexity of its rhythmic function is linked to its structure divided into compartments (33). In the seminiferous tubule there is a spermatogenic wave that travels along in length to determine the timing of the commitment of spermatogonia to differentiate, the phases of meiotic division, and the rate of differentiation of the post-meiotic germ cells (33). Leydig cells, localized in the interstitial space, produce steroid hormones to ensure spermatozoa maturation and the sexual characteristics (33).

Several lines of evidence exist suggesting that disruption of mechanisms involved in CR may interfere with male fertility.

### Mutations of Clock Genes and Male Infertility

Clock proteins have been reported to be expressed in male germ epithelium. In details, BMAL1 localizes mainly in Leydig cells but it can also be found in the nucleus and cytoplasm of germ cells and CLOCK was observed having a higher expression level in the cytoplasm of round spermatids (34).

Both pre-clinical and clinical evidence suggest the involvement of clock genes in the pathophysiology of male infertility.

**Table 1** summarizes evidence from pre-clinical animal studies.

**Table 1:** Findings from pre-clinical studies investigating the role of clock genes on spermatogenesis.

Author, year	Pre-clinical model	Clock genes evaluated	Findings
Morse et al. (35)	*Clock* mutant male mice	*Per1*	*Per1* expression is not altered in testes from *Clock* mutant mice
Alvarez JD et al. (36)	*Bmal1* KO male mice	*Bmal1*	Impaired steroidogenesis in *Bmal1* KO mice. Reduced average seminiferous tubule diameter and the sperm counts in *Bmal1* KO mice.
Peruquetti RL et al. (34)	*Bmal1* KO male mice *Clock* KO male mice	*Bmal1* *Clock*	Alteration in the structure of chromatoid body of the spermatids of *Bmal1* KO and *Clock* KO mice
Liang X et al. (37)	*Clock* KD male mice	*Clock*	Smaller litter size, lower *in vitro* fertility rate, lower blastula formation rate, and lower acrosin activity in *Clock* KD male mice.

KD, Knock-down; KO, Knockout.

Alvarez et al. used *Bmal1* knockout (KO) mice in order to clarify the role of this circadian clock protein in the fertility process and its role in testosterone production (36). Authors found that male *Bmal1* KO mice were infertile and had lower testosterone and higher luteinizing hormone (LH) serum concentrations, compared to wild-type mice thus suggesting a defect in testicular Leydig cells. Testes and other steroidogenic tissues of *Bmal1* KO mice exhibited reduced expression of steroidogenic genes. Expression of the *steroidogenic acute regulatory protein* (*StAR*) gene and protein, which regulates the rate-limiting step of steroidogenesis, was decreased in testes from *Bmal1* KO mice. Microscopically, testes from *Bmal1* KO mice exhibited reduced average seminiferous tubule diameter (by approximately 20%) and the sperm counts were reduced by approximately 70%. Homozygous *Bmal1* KO mice were unable to breed with each other (36).

Peruquetti et al. analyzed the role of two genes (*Clock* and *Bmal1*) in the chromatoid body. This cytoplasmic organelle plays a crucial role in RNA post-transcriptional and translation regulation during the germ cell differentiation. They detect an alteration in the structure of chromatoid body of the spermatids of *Bmal1* KO and *Clock* KO mice (34).

Liang et al. knocked down the *Clock* gene expression in the testes of male mice and determined its effect on fertility. Authors recorded lower *in vitro* fertilization rate, lower blastula formation rate, and a lower acrosin activity in the *Clock* Knockdown sperms, as well as a delay in dispersing cumulus cells. These results demonstrate that acrosin activity could be regulated by *Clock* and that *Clock* contributes to the regulation of male fertility and blastula formation (37).

Cheng et al. further corroborated these observation: they verified that acrosin activity and *in vitro* fertilization rate were reduced in SERPINA3K-treated sperm, producing a pattern of phenotypes very similar to that previously observed in the *Clock* knockdown sperm (38).

Unfortunately, the circadian expression of clock genes in testis tissue remains controversial.

Morse et al. investigated the role and the expression of *Per1* in the mice testis, reporting a constant expression of the gene during the 12-h light and 12-h dark cycles. Moreover, the levels of *Per1* proteins were constant during the day (35).

Human studies also provided evidence about the involvement of clock genes in male infertility.

Zhang et al. demonstrated, for the first time, an association between *CLOCK* genetic variants and altered semen quality in a human population with idiopathic infertility (39). In details, authors investigated the association between genetic variants of *CLOCK* and semen quality in humans. Authors examined three Single-Nucleotide-Polimorfism (SNP) of the *CLOCK* gene, (i.e. rs3749474, rs1801260 and rs3817444) to assess the association between these variants and semen quality in men with idiopathic infertility. The results indicated a strong association between the C allele carriers (CC or CT) of rs374947 and significantly reduced semen volume and sperm number per ejaculate. Moreover, associations between the A allele carriers (CA or AA) of rs3817444 and significantly reduced semen volume as well as between both the rs3749474 CC genotype and rs1801260 TC genotype and significantly decreased sperm motility were found (40).

### CD and Reproductive Hormones

Circulating levels of male sexual hormones are modulated by circadian clock, light exposure, and sleep duration. The circadian pattern of LH and testosterone have been widely investigated in healthy males. In healthy young men, serum testosterone concentrations rise with sleep onset, reaches the peak during the first REM episode, remains stable until awakening, and then rapidly declines (41). Sleep-related elevations in LH have been also reported. Total sleep time and durations of stage 2 and REM have been reported to be positively related to morning testosterone levels (41). Zhang et al. found a significant correlation between sleep duration (measured by actigraphy) and follicle stimulating hormone (FSH) levels as well as by rapid eye movement sleep and FSH in healthy young men (42).

Total sleep deprivation or sleep restriction have been shown to impair the secretory activity of the pituitary-testis axis.

Leproult et al. investigated the effect of 1 week of sleep restriction to 5 hours per night (a condition experienced by at least 15% of the US working population) on testosterone levels in young healthy men and found a daytime decrease of testosterone levels by 10% to 15% (43).

Schmid et al. aimed to discriminate the effects of sleep duration and sleep timing on serum concentrations of LH and testosterone (44). They failed to find differences in terms of serum LH and testosterone concentrations between patents with sleep time restriction to 4 h for two consecutive nights (bedtime, 02:45 -07:00 h) and a control condition of 8 h regular sleep (bedtime, 22:45-07:00 h). However, total sleep deprivation and 4·5 h of sleep restricted to the first night-half (bedtime, 22:30-03:30 h) markedly decreased morning testosterone concentrations (44).

Interestingly, testosterone level has been reported to recover basal concentrations after one night of recovery sleep. However, extending sleep duration by approximately 1.2 h/night over six nights has minimal effects on hormonal responses to total sleep deprivation (41).

Yoon et al. investigated the levels of urinary LH in normal young men aged between 19 and 30 years following early morning light exposure (05:00 – 06.00). They found that LH excretion was increased 69.5% after bright light exposure but was not changed by placebo light exposure (45).

Despite previous results, Chen et al. failed to find significant association between sleep duration and reproductive hormone levels (follicle-stimulating hormone, LH, estradiol, progesterone, testosterone, and prolactin (46).

### CD and Spermatogenesis in Humans

Evidence about the influence of CD on spermatogenesis in humans derive mainly from studies about the relationship between sleep duration/architecture and sperm parameters (**Table 2**).

**Table 2:** Findings from clinical studies investigating the association between sleep duration/quality and semen parameters.

Author, year	Study population	Findings
Jensen et al. (47)	Healthy men (n=953)	Inverse U-shaped association between sleep disturbance score and sperm concentration, total sperm count, percent morphologically normal spermatozoa (poorer semen quality in men with a sleep score below or above 11– 20).
Eisenberg et al. (48)	Men during preconception period (n=456)	No significant association between night or shift work and semen parameters
Chen et al. (46)	Healthy men (n=796)	Inverse U-shaped association between sleep duration and semen volume and total sperm count.
Vigano et al. (49)	Inferile men (n=382)	Negative association between sleep quality (difficulty in initiating sleep or lying awake most of the night) and sperm parameters concentration or motility.
Liu et al. (50)	Healthy men (n=1346)	Lower total sperm count in rotating shift workers.
Shi et al. (51)	Healthy men (n=328)	Decreased sperm concentration in short (< 4.7h) sleepers. Higher sperm DNA fragmentation index in patients with irregular sleeping habits.
Pokhrel et al. (52)	Healthy men (n=1101)	No association between sleep duration and sperm parameters.
Chen et al. (53)	Healthy men candidates for being sperm donor (n=842)	Association between short (<6 h) or long (>9 h) sleep duration and reduced sperm motility. Association between bad sleep quality (total Pittsburgh Sleep Quality Index [PSQI] score >5.0) and lower total sperm count, total motility, and progressive motility.
Du et al. (54)	Infertile men (n=970)	Negative correlation between the general quality of sleep and total motility, progressive motility, concentration, total sperm number and normal morphology.
Green et al. (55)	Healthy men (n=116)	Positive correlation between sleep duration and total sperm count progressive motility. Negative correlation between the usage of digital devices at night and total motility, progressive motility, concentration. Positive correlation between the usage of digital devices at night and immotile sperm.
Hvidt et al. (56)	Infertile men (n=104)	Lower semen quality in short (7.0–7.49 h) and very short (< 7.0 h) sleepers. Association between late (≥11:30 PM) bedtime and reduced semen quality. No association between sleep quality and semen quality.

Zhang et al. found a significant correlation between sleep duration (measured by actigraphy) and testis volume (42).

Chen Q. et al. recorded an inversed U-shaped association between the entire sleep duration and two of the semen parameter (semen volume and total sperm count) with 7.0-7.5 h/d as the “turning point”: for those whose sleep duration was below 7.0 h/d the semen parameters increased with longer sleep duration; but the semen parameters decreased, with longer sleep duration, for those whose sleep duration was over 7.5 h/d (46).

Similarly, Chen H-G. et al. investigated association between sleep duration and quality of the semen samples (53). Authors found a better quality of the semen (sperm volume, concentration and total count) in men who slept between 8.0 and 8.5 hours per day (53). Men who slept less than 6.0 h/day and higher than 9.0 h/day had lower sperm volume of 12% and 3.9%, respectively. Men who slept less than 6.0 h/day had lower total and progressive sperm motility of 4.4% and 5.0%, respectively. Moreover, men reporting poor sleep quality (total Pittsburgh Sleep Quality Index [PSQI] score >5.0) had lower total sperm count, total motility, and progressive motility of 8.0%, 3.9%, and 4.0%, respectively (53).

Jensen et al. demonstrated an inverse U-shaped association between sleep disturbance score and sperm concentration, total sperm count, percent morphologically normal spermatozoa (47).

Du et al. recorded a negative correlation between the general quality of sleep and several semen parameters (total motility, progressive motility, concentration, total sperm number and normal morphology) although semen volume and reproductive hormones were not markedly altered (54).

Green et al. recorded a positive statistically significant correlation between sleep duration and some semen parameters (total sperm count and progressive motility) (55). Additionally, authors recorded for the first time a negative significant correlation between the usage of digital devices, especially smartphones, at night, and sperm quality (total motility, progressive motility, concentration) and a positive statistically significant correlation between the usage of these devices and immotile sperm. It was hypothesized that the pathological mechanism underlying this phenomenon was the alteration in melatonin secretion induced by the short wavelength light emitted from the screens of these electronic devices (55). These results were corroborated by several studies, that report an association between elevated melatonin levels and oligospermia or azoospermia in men (57, 58).

Liu et al. compared sperm parameters between rotating shift workers (RSW) and permanent shift workers (PSW). They recorded a significantly lower total sperm count in RSW, compared to PSW. RSW was associated with higher risk of low total sperm count, after adjusting for age, education level, average monthly household income, abstinence period, sampling time point, tobacco smoking, alcohol drinking and body mass index (50).

Liu et al. conducted the same analyses on a cohort of male undergraduates in order to identify semen quality differences associated with non-work-related CD between school days and days off. Total sperm count in men with less than 0.5 h of CD was significantly higher compared to men with more than 2 h of CD (50).

Hvidt et al. found that early bedtime (< 10:30 PM) was more often associated with normal semen quality compared with both regular (10:30 PM-11:29 PM) and late (≥11:30 PM) bedtime. Similarly, regular sleep duration (7.5–7.99 h) was more often associated with normal semen quality than both short (7.0–7.49 h) and very short (< 7.0 h) sleep duration (56).

Of note, Shi et al. found significant associations between sperm DNA fragmentation index and irregular sleep habits (51).

Despite previous evidence, other studies failed to find significant association between sleep duration and or quality and sperm parameters (48, 52).

## Discussion

The relationship between disruption of CR and male infertility is supported by several pre-clinical and clinical lines of evidence. First, experimental manipulation or spontaneous mutations of clock genes, such as *Bmal1* and *Clock* negatively interfere with fertility pathways in both pre-clinical models and humans. Second, alterations of CR due to altered sleep duration and/or impairment of sleep architecture may negatively interfere with circulating levels of reproductive hormones and semen parameters in humans. Unfortunately, the current level of evidence is still low, and findings are often controversial due to heterogeneity in terms of study design, study populations, and standardization of measurements. Therefore, further studies are needed to further elucidate the interactions between CR, CD, and male infertility. In details, the impact of the duration of CD, genetic predisposing factors, as well the reversibility of these alterations deserve further investigations. Based on available evidence, clinicians facing infertile males should discuss, in everyday clinical practice, the potential detrimental ole of reduced sleep duration, altered sleep architecture or exposure to artificial light at night on reproduction.

## Author Contributions

Conception and design: FF, NL, and MCr. Acquisition of data: LS, LL, GF, and LC. Analysis and interpretation of data: MS, DA, and LN. Drafting of the manuscript: SM, GCa, CT, and GS. Critical revision of the manuscript for important intellectual content: MCr, GCe, MCa, RR, FF, FM, VM, and NL. Supervision: FF, NL, and MCr. All authors contributed to the article and approved the submitted version.

## Conflict of Interest

The authors declare that the research was conducted in the absence of any commercial or financial relationships that could be construed as a potential conflict of interest.

## Publisher’s Note

All claims expressed in this article are solely those of the authors and do not necessarily represent those of their affiliated organizations, or those of the publisher, the editors and the reviewers. Any product that may be evaluated in this article, or claim that may be made by its manufacturer, is not guaranteed or endorsed by the publisher.
